# The Lived Experiences of Highly Educated Internationally Educated Nurses Transitioning to Practice in Canada

**DOI:** 10.1177/23779608261465162

**Published:** 2026-06-26

**Authors:** Emilene Reisdorfer, Mona Haimour, Amina Regina Silva, Mary Asirifi, Margaux Papadopoulos

**Affiliations:** 1 Department of Professional Nursing and Allied Health, Faculty of Nursing, 3151 MacEwan University, Edmonton, AB, Canada; 2 Department of Integrative Nursing Systems, Faculty of Nursing, 3151 MacEwan University, Edmonton, AB, Canada; 3 7497 Department of Nursing, Brock University, St. Catharines, ON, Canada; 4 Department of Nursing Foundations and Science, 3151 Faculty of Nursing, MacEwan University, Edmonton, AB, Canada; 5 3146 Northern Addiction Center, Recovery Alberta, Grande Prairie, AB, Canada, Edmonton, AB, Canada

**Keywords:** international nurses, graduate education, professional practice, Canada

## Abstract

**Background:**

Highly Educated Internationally Educated Nurses (HE IENs) face unique challenges when transitioning into Canadian nursing practice. Despite their advanced qualifications, they often encounter systemic barriers, including credential recognition issues, licensing complexities, cultural adaptation, and discrimination.

**Objective:**

To explore the lived experiences of HE IENs transitioning to practice in Canada, identify key challenges and facilitators, and offer recommendations to support their integration and retention.

**Methods:**

HE IENs were recruited through purposive and snowball sampling. Data was collected via semi-structured narrative interviews and analyzed using Deductive Qualitative Analysis (DQA), which combines deductive and inductive coding.

**Results:**

14 participants shared that graduate education was both an asset and a barrier. While it enhanced confidence and academic preparedness, it was often undervalued by employers and regulatory bodies. Participants reported being labelled “overqualified,” facing opaque licensing processes, and experiencing racism and cultural exclusion. Many concealed their credentials to access employment or pursued Canadian degrees to gain legitimacy. Facilitators included peer networks, bridging programs, and personal resilience. Despite systemic challenges, participants remained committed to their profession and demonstrated adaptability and advocacy.

**Conclusions:**

HE IENs bring valuable expertise but face significant integration barriers in Canada. Their experiences highlight the need for inclusive, transparent systems that recognize international credentials and support equitable workforce participation. Tailored strategies—such as mentorship, centralized information platforms, credential recognition, and alternative registration pathways—are essential to improve HE IENs’ integration and retention.

## Introduction

Internationally Educated Nurses (IENs) play a crucial role in addressing global healthcare demands by bringing diverse perspectives, cultural competence, and specialized skills to nursing practice (Balante et al., 2021; Miyata, 2023). An IEN is an individual who has pursued nursing education and training outside of the country where they intend to practice. Motivated by factors such as better compensation, career growth opportunities, or personal reasons, IENs migrate to new countries to pursue employment in nursing (Miyata, 2023).

Despite the essential role of IENs in sustaining workforce numbers and advancing equity and patient-centred care on a global scale, this population often faces marginalization within the healthcare profession due to systemic barriers that hinder their full integration into the workforce (Newton et al., 2012). Research has highlighted various factors contributing to the global marginalization of IENs, including challenges related to licensure, accreditation, and recognition of foreign qualifications (Covell et al., 2014). The process of obtaining licensure in a new jurisdiction can be arduous and complex, involving assessments of educational credentials, language proficiency exams, and clinical competency evaluations (Cheng et al., 2013). Additionally, IENs may encounter limited access to support networks, mentorship programs, and professional development opportunities, which are essential for their successful integration (Higginbottom, 2011). Discrimination based on nationality, ethnicity, or accent further exacerbates feelings of marginalization and exclusion among IENs (Salami et al., 2018). Socioeconomic challenges, such as financial strain and housing insecurity, also contribute to the marginalization experienced by IENs (Salami et al., 2018).

### Review of the Literature

In the context of workforce optimization and improvement, some IENs possess advanced or specialized education and training in nursing, distinguishing them as highly educated professionals. Some individuals may hold advanced degrees, such as a Master’s or Doctorate in nursing or a related field, along with specialized certifications in areas like nursing practice, education, or research. This advanced academic background underscores their expertise, but may also influence their transition to practice and licensing processes, as their credentials might not be valued or recognized in the new country (Salami et al., 2018).

Highly educated IENs (HE IENs) face additional challenges as they transition to practice, including reconciling their advanced academic background with the practical realities of the healthcare environment in their new jurisdiction. HE IENs may also face difficulties translating their theoretical knowledge into clinical practice or may encounter resistance from colleagues who perceive them as overqualified (Miyata, 2023). Additionally, navigating the intricacies of the healthcare system, understanding local protocols and practices, and adapting to cultural nuances can pose significant hurdles during the transition phase (International Council of Nurses, 2019; Salmond & Echevarria, 2017).

Existing research has extensively documented the challenges faced by IENs, including systemic barriers related to licensure, credential recognition, discrimination, and limited institutional support within host countries (Covell et al., 2014; Salami et al., 2018). These studies have been instrumental in advancing understanding of workforce integration issues affecting IENs as a broad population. However, much of this literature treats IENs as a relatively homogeneous group, offering limited attention to how variations in educational attainment shape nurses’ transition experiences. What remains insufficiently understood is how HE IENs, many of whom hold master’s or doctoral degrees and bring extensive academic, research, and leadership expertise, experience and navigate the transition to practice in Canada. Specifically, there is a lack of empirical research exploring how advanced qualifications influence professional identity, expectations of recognition, experiences of deskilling, and patterns of adaptation during transition.

Meleis’ Transition Theory provides a useful theoretical lens for understanding the transitions of HE IENs as they navigate the complex process of entering nursing practice in a new country. Transition theory conceptualizes transition as a multifaceted process characterized by changes in roles, identities, relationships, and patterns of behaviour, all of which require time, support, and effective coping strategies to achieve healthy outcomes (Meleis, 2017; Meleis et al., 2000). HE IENs frequently experience multiple and overlapping transitions, including professional, organizational, cultural, and geographic transitions, which can occur simultaneously and intensify vulnerability during the integration process.

This study sought to explore these experiences, clearly identify the challenges and opportunities faced by HE IENs during their transition, and provide focused recommendations for strategies and programs to support their successful integration into Canadian nursing practice. The central research question guiding this study was: What are the lived experiences of HE IENs transitioning to practice in Canada?

This study foregrounded the distinct transition experiences of HE IENs, an underexamined yet critical segment of the nursing workforce. The findings demonstrated how advanced qualifications influence expectations, professional identity, and experiences of recognition or marginalization within Canadian healthcare systems. The study offered evidence to inform more equitable regulatory processes, tailored transition supports, and alternative workforce pathways that better align with the expertise of HE IENs. These contributions are particularly timely given ongoing nursing workforce shortages and the need to optimize the integration and retention of highly skilled nurses.

## Material and Methods

This qualitative study employed Meleis’s Transition Theory to support the data collection and analysis. This theory offers a valuable lens through which to explore and contextualize these challenges. This framework provides a comprehensive understanding of transitions as dynamic processes that involve movement from one state to another (Meleis, 2010). Transitions are recognized as significant life changes that encompass shifts in roles, relationships, routines, and environments (Meleis et al., 2000). Situational transitions refer specifically to changes in circumstances, such as moving to a new country or adjusting to a different professional environment.

The theory conceptualizes transitions through three key components: the transition itself, the conditions that influence the transition, and the outcomes of the transition (Meleis, 2017). In the context of HE IENs transitioning to practice in a new environment, the Transition Theory can be applied as follows:

-** Transition:** This theory component focuses on the actual process of moving from one country to another.
-** Conditions:** The conditions that influence the transition refer to the internal and external factors that shape individuals’ experiences during the transition process.
-** Outcomes:** The outcomes of the transition refer to the consequences of the transition process.

### Sampling Strategy

The participants included in the study were IENs who possessed advanced degrees, specifically either a Master’s or a PhD in Nursing, who obtained at least one graduate degree from their home countries or other foreign countries, excluding Canada. Participants were required to have initiated the relicensing process after 2014 and completed it before participating in the study. Additionally, prospective participants had to be fluent in English or French. Participants were excluded from the study if they had not completed the re-licensing process, were not living in Canada, or had completed all their graduate education in Canada.

Participants were selected utilizing purposive and snowball sampling techniques to ensure they met the specified inclusion criteria. Purposive sampling was employed to choose individuals who fulfilled the requirements necessary to achieve the study’s objectives. Snowball sampling was incorporated to enhance participant recruitment and reach individuals and communities that might have been overlooked by purposive sampling alone (Naderifar et al., 2017).

A monetary reimbursement was offered as compensation for the participants’ time and willingness to share their experiences. To advertise the study, a bilingual poster (in English and French) was created to ensure accessibility and inclusivity across Canada. This poster was disseminated through various online platforms to effectively reach our target audience.

### Data Collection

The interviews were conducted on an individual basis and were held virtually through the Microsoft Teams platform. The data collection took place from June to October, 2024.

The semi-structured interview guide contained probing questions that were utilized to guarantee that the objectives of the study would be met. Further questions were added to guide the participants during the interview (Supplementary File 1). Examples of questions aligned to the Transition Theory (Meleis, 2017) are:

**Conditions**: Provide details about your professional background in your home country; do you have a professional support network in Canada (for instance, other IENs, institutions, colleagues, etc)? Can you tell me about the type of professional support you have received since your arrival?

**Transition:** Provide some background information about your nursing education and professional experience; talk about your decision to move to Canada; Talk about the transition to practice in Canada and any factors that influenced this decision.

**Outcomes**: Talk about your plans for the future; How was the job searching process once you received your license?

Experienced female researchers led the interviews, while the research assistants supported data collection and revised the transcripts. The researchers were either Master’s or PhD prepared and had previous training and expertise in the research process. Interview assignments were made with consideration of any prior personal relationships between researchers and participants, in order to minimize bias and ensure the integrity and neutrality of the data collection process.

Data saturation was achieved when no new insights or perspectives emerged from subsequent interviews. As researchers conducted and analyzed each interview, they continuously compared the data to identify recurring patterns and concepts.

### Data Analysis

The data was analyzed using the Deductive Qualitative Analysis (DQA) outlined by Fife and Gossner (2024). This approach involved applying the predetermined frameworks of the Transitions Theory - Situational Transitions (Meleis, 2017) to guide the coding and interpretation of the participants’ testimonials. Researchers operationalized the theories by generating sensitizing constructs and key concepts from the guiding theory to guide the initial data analysis.

DQA is a qualitative methodology used to systematically evaluate, refine, and potentially expand existing theory by analyzing empirical data through both deductive and inductive lenses. Rather than generating theory from scratch, DQA begins with a guiding theory that is operationalized as sensitizing constructs to direct the analysis. Researchers then examined the data for evidence that supports, contradicts, refines, or expands this initial theory, while deliberately engaging in inductive analysis to avoid confirmation bias (Fife & Gossner, 2024). Deductive Qualitative Analysis (DQA) was selected as the methodological approach because it enabled a theory-driven examination of HE IENs’ transition experiences while remaining sufficiently flexible to capture unanticipated aspects of participants’ narratives. This approach was particularly suited to the study’s aim of both applying and extending Meleis’ Transition Theory within a contemporary Canadian context.

Early data analysis involved two researchers independently immersing themselves in the data, using sensitizing constructs derived from the guiding theory as initial deductive codes. In this phase, a deductive approach was used to apply sensitizing constructs derived from Meleis’ Transition Theory as initial coding categories, guiding the organization of the data. Concurrently, inductive coding was undertaken to identify emergent concepts and experiences not fully explained by the theoretical framework (Fife & Gossner, 2024). The researchers met to discuss their findings, and if intercoded agreements were not achieved, a third researcher revised and made the final decision.

Next, middle analysis shifted the focus toward theme development and deeper engagement with four types of evidence: supporting, contradicting, refining, and expanding (Fife & Gossner, 2024). In this phase, the analysis shifted toward an integrative process that refined the relationship between deductive and inductive findings. Researchers reviewed the data to determine how well the sensitizing constructs held up, whether they needed revision, or if new constructs should be added. In the process, codes were grouped into themes according to the topic and theoretical relationships. A revised codebook was used to systematically track these changes and support the evolving understanding of the phenomenon (Fife & Gossner, 2024).

Lastly, theorizing occurred throughout the study and culminated in the interpretation of evidence and the proposal of revisions to the initial theory. The final phase emphasized the synthesis of deductive and inductive insights to extend the guiding theory. Researchers integrated supported constructs, refined those that required adjustment, and incorporated new inductively derived themes. Theorizing also included critical reflection on the assumptions underlying the theory and consideration of alternative theoretical lenses (Fife & Gossner, 2024).

To enhance transparency and analytic rigour, the DQA process unfolded in a clearly defined sequence. First, Meleis’s Transition Theory was operationalized into sensitizing constructs aligned with situational transitions, conditions, and outcomes, which guided the initial deductive coding. Simultaneously, inductive coding was deliberately employed to capture experiences and meanings not adequately explained by the existing theoretical framework. Through independent coding, analytic comparison, and consensus discussion among researchers, codes were progressively refined and organized into themes and subthemes. In the middle analytic phase, data were systematically examined for evidence that supported, contradicted, refined, or expanded the guiding theory. The final theorizing phase integrated these analytic insights to both validate and extend Transition Theory in the context of HE IENs, ensuring that findings were grounded in participants’ narratives while remaining theoretically coherent.

### Trustworthiness and Credibility

To ensure the study’s trustworthiness, the researchers employed strategies such as documenting the research process, taking notes during the interviews, and recording the data collection process. Each virtual interview was conducted through Microsoft Teams, and the data was stored on encrypted computers. The interviews were transcribed using Microsoft Teams, and the research assistants verified each transcript for accuracy. The complete data analysis was done through NVivo. To ensure the credibility of the data, all interviews were fully read and double-checked by the PI and research assistants. Findings were also compared to field notes.

The audit trail in this paper was implemented through several explicit steps. The authors maintained detailed documentation of the research process, including notes taken during interviews and records of data collection procedures, ensuring that all analytic decisions could be traced back to the original data. All interviews were conducted via Microsoft Teams, recorded, and then transcribed, with research assistants verifying each transcript for accuracy, which created a clear record from raw data to analyzed text. The use of NVivo software further supported the audit trail by providing a systematic and trackable platform for coding, organizing, and managing data throughout the analytic process. Finally, the structured Deductive Qualitative Analysis (DQA) approach, with its clearly defined phases (early analysis, middle analysis, and theorizing), contributed to the audit trail by outlining a transparent sequence of analytic steps, allowing readers to follow how themes and theoretical insights were developed from the data.

Reflexivity in this study was demonstrated through the authors’ explicitly structuring the data collection process to minimize bias by considering any prior relationships between researchers and participants when assigning interviews, thereby reducing the potential for pre-existing dynamics to shape responses. As a final step to guarantee rigour, triangulation was performed by the authors by comparing field notes, perceptions and perspectives, use of a third researcher to resolve discrepancies in data analysis, as well as collaborative team discussions to ensure the researchers’ biases were identified and mitigated from the study.

Thick description was also utilized by the authors. Detailed accounts of participants’ lived experiences, supported by contextualized narratives and direct quotations from interviews were included. The inclusion of verbatim excerpts allowed readers to understand the meanings participants attributed to their experiences, such as navigating credential recognition, workplace discrimination, and identity transitions within the Canadian healthcare system. Additionally, the participants’ backgrounds were described in depth—including countries of origin, years of experience, professional roles, and migration pathways—which helped situate the findings within a broader social and professional context. The presentation of themes and subthemes, along with detailed explanations and illustrative examples, further enriched the interpretation of the data.

### Ethical Considerations

Ethical approval was obtained from the Research Ethics Board at MacEwan University, File No: 102315 on May 9, 2024. The participants were informed of the purpose of the research and the protection of their rights, and signed the Informed Consent prior to the start of the interview. Participants were informed that they could withdraw from the study at any point without any consequences.

## Results

The cohort of HE IENs was composed of 14 individuals, with the interview duration time ranging from 30 to 90 minutes. The interviews were conducted from June to November 2024. The group was predominantly female, ranging in age from 31 to 55 years. These nurses came from countries including Nigeria, Rwanda, Pakistan, the Philippines, Brazil, Jordan, and India. Their ethnic identities span Asian, African, South Asian, Cordilleran, Latina, Arab, and Brazilian backgrounds. The participants immigrated to Canada through family sponsorship, a postgraduate work permit, or a skilled professional program. The average time since immigrating to Canada was 8.5 years.

With nursing experience ranging from nine to 34 years, and half holding master’s degrees, while the other half possess additional PhD, these professionals bring a depth of knowledge and leadership potential. Many obtained their initial nursing degrees abroad and pursued advanced education in countries such as the United States of America, the United Kingdom, Japan, and Canada. Their first Canadian RN licenses were issued between 2006 and 2024, across provinces like Ontario, Alberta, and New Brunswick. Some were required to complete bridging programs, while others navigated the licensing exams to meet regulatory standards.

Despite their qualifications, many continued to face employment barriers, with some working full-time, others part-time or on temporary contracts, and a few who were still seeking opportunities. Of the participants, seven were working in clinical practice, six were in academia, and one was not currently working. Their areas of clinical practice spanned over med-surg, intensive care unit (ICU), community health, mental health, oncology, neonatal care, and post-secondary education. Many remained licensed in their countries of origin, underscoring their ongoing global ties.

Participants’ advanced academic qualifications significantly shaped their expectations of professional recognition, role alignment, and career progression in Canada. Rather than conferring advantage, graduate degrees frequently intensified experiences of deskilling, role strain, and identity disruption, as HE IENs encountered being labelled “overqualified,” advised to conceal credentials, or required to repeat foundational training despite extensive clinical, academic, and leadership experience. These findings highlighted how advanced education alters the nature of transition by reshaping professional identity, amplifying dissonance between expectations and realities, and creating unique forms of marginalization that extend beyond those commonly described for IENs. Centering graduate education as a defining condition of transition highlighted challenges and adaptive strategies unique to HE IENs, reinforcing the need for differentiated analysis and targeted regulatory, institutional, and workforce integration supports.

### Themes and Subthemes

The DQA process began with the identification of main sensitizing constructs and key concepts based on the framework of Transition Theory, specifically, the concept of Situational Transition (Meleis, 2017). These preliminary sensitizing constructs served as a guiding structure for the early stages of data analysis, allowing the researchers to systematically code and interpret the interview transcripts. As the analysis progressed and the researchers engaged more deeply with the data, eight additional sensitizing constructs emerged inductively from the participants’ narratives, reflecting nuances not captured in the original framework.

During the middle analysis phase of a DQA process, the sensitizing constructs and synthesizing findings were revised into coherent themes and subthemes. Four themes from the initial framework remained valid and were supported by the data. However, the sensitizing constructs were conceptually overlapping or closely related, so they were reorganized and grouped together as 12 subthemes to better reflect their interconnections and improve the clarity and coherence of the theoretical model (Table 1).

**Table 1.** Middle Analysis Themes and Subthemes

Themes	Subthemes (middle analysis)	Early analysis
Change Triggers and Properties of Transition	Influence of graduate studies on the decision to immigrate	- Does having a master’s degree/PhD influence your decision to move to Canada?
	Nursing Education and Professional Experience outside Canada	- Initial nursing education and professional experience before you arrived in Canada
		- Submitting graduate transcripts
		- The decision to move to Canada
Transition: Facilitators and Inhibitors	Influence of graduate studies on transition to practice	- Advocacy
		- How does having a master’s degree/PhD influence your transition to practice?
		- Level of education
		- Supports related to the level of education
		- Professional growth and development as related to educational level
		- Retention in the nursing workforce
	Regulatory Requirements and Educational Gaps	- Bridging Education
		- Competency
		- Educational gap (bridging)
		- Healthcare system structures in their new country
		- Language proficiency
		- Licensing challenges
		- Mentorship
		- Regulatory requirements
	Transition to Canadian practice	- Current work experience
		- Transition to practice in Canada and any factors that influenced this experience
		- Challenges encountered during the transition process
	Personal supports and challenges	- Personal challenges
		- Social support networks
		- Opportunities/facilitators you encountered during the transition process
	Racism and Discrimination	- Cultural competence
		- Cultural safety (racism and discrimination)
Patterns of Response: Progress and Outcomes	Expectations regarding a graduate degree	- Expectations regarding a graduate degree
		- Professional growth and development as related to educational level
	Overall wellbeing	- Job satisfaction
		- Overall well-being
Recommendations for HE IENs	Recommendations	- Recommendations

The theorizing phase of the DQA process occurred iteratively throughout data analysis, as sensitizing constructs, subthemes, and themes were identified and revised through both deductive and inductive approaches. A detailed account of the final theorizing process is provided in the description below.

#### Change Triggers and Properties of Transition

Graduate education played a significant role in triggering changes in the immigration decisions of HE IENs, shaping their aspirations for research, teaching, and professional advancement in Canada. These academic experiences often inspired nurses to seek environments where their expertise would be more valued; however, personal circumstances, such as family needs or burnout, also influenced their decisions.

##### Influence of Graduate Studies on the Decision to Immigrate

The decision to move to Canada was often influenced by a combination of professional aspirations and personal circumstances. Some nurses were motivated by the desire for better opportunities, international exposure, or improved quality of life for their families. One participant explained, *“I came to Canada on a student visa to do my PhD… I never had the intention of staying” (Participant 3).* These decisions were sometimes spontaneous or shaped by life events, such as health crises or burnout, prompting a reevaluation of priorities and career paths.

Graduate studies, such as a master’s degree or PhD, had a varied influence on HE IENs’ decisions to immigrate to Canada. For some, these qualifications were seen as assets that could ease the immigration process or enhance professional opportunities. One participant noted, *“It was an advantage for me to have a master’s degree because I have been in schools” (Participant 2),* suggesting that prior academic exposure helped with exam preparation and understanding Canadian standards.

Graduate education also influenced the decisions of some nurses to immigrate, particularly those motivated by research opportunities or a desire to teach. For example, one nurse explained that her background in research and clinical inquiry inspired her to move to Canada, where she felt her work would be more valued and applicable. Some nurses were motivated by the potential to continue academic or research careers in Canada. One participant shared, *“I really wanted to come and further develop my career as a researcher in nursing” (Participant 8),* illustrating how graduate education influenced long-term aspirations and the desire to continue on their chosen path.

##### Nursing Education and Professional Experience Outside Canada

The participants highlighted the depth and diversity of nursing education and professional experience they bring, as well as the challenges they face in having that experience recognized in Canada. Participants consistently described extensive and varied professional backgrounds before immigrating. Many had worked in specialized areas such as ICU, maternity, oncology, and mental health, and held roles ranging from bedside nurses to assistant professors and program directors. One nurse shared, *“I taught management and leadership, women’s health, triage, urgent care, critical care… I did my PhD in the meantime, working in two places” (Participant 11),* illustrating the high level of expertise and multitasking involved in their careers. These experiences were often accompanied by advanced degrees, including master’s and PhDs, which were pursued alongside demanding clinical and academic roles.

#### Transition: Facilitators and Inhibitors

The transition of HE IENs into Canadian practice was shaped by a complex interplay of facilitators and inhibitors. Graduate education served as both a strength and a barrier—enhancing confidence, academic preparedness, and advocacy skills, yet often being undervalued in employment contexts. Institutional inhibitors included opaque regulatory processes, inconsistent licensing standards, and rigid language requirements, which created confusion and emotional strain. Cultural adaptation and experiences of racism further hindered integration, with many nurses feeling compelled to suppress aspects of their identity. However, facilitators such as peer networks, bridging programs, family support, and community connections provided emotional resilience and practical guidance.

##### Influence of Graduate Studies on Transition to Practice

The results highlighted how graduate education influenced the transition of HE IENs to practice in complex and often contradictory ways. Three main ideas emerged: graduate studies provide academic and personal advantages, they create unexpected barriers in employment and recognition, and they foster a sense of advocacy and resilience.

First, graduate studies strengthened nurses’ confidence and academic preparedness, even if this did not always translate directly into practice opportunities. One nurse explained that their master’s degree helped with professional exams: *“When I was preparing for my NCLEX exam, … I found a topic that I had encountered in my master’s” (Participant 2).* Others described how advanced education sharpened their communication skills and research mindset, equipping them with the tools to adapt to Canadian systems despite cultural and linguistic barriers. These academic benefits often translated into greater self-assurance, even if institutional recognition lagged behind.

Despite the advantages, many participants reported that higher education was undervalued or even detrimental in the job market. Several nurses described being told they were “overqualified” for positions or that:

“Even if you have your master's and your PhD, it won’t even help you get in the market even as a licensed practical nurse or healthcare aid” (Participant 4).

Others concealed their graduate degrees when applying for entry-level roles because *“I don’t think they (employers) care about it at all… it was just points in your immigration paper” (Participant 7)*. This mismatch between qualifications and employment expectations left many feeling frustrated and, at times, discouraged. The nurses explained that they would conceal their graduate degrees when applying for certain positions in order to increase their chances of getting a job. One participant shared that she received advice to remove all her academic credentials from her resume to increase her chances of finding a job *(Participant 12)*.

Despite their qualifications, many participants encountered barriers in transferring their credentials and having their education recognized. Submitting graduate transcripts did not always result in meaningful progress and was often insufficient to bridge the educational gaps identified by regulatory bodies. One participant stated, *“I sent my master’s curriculum… but it wasn’t sufficient still” (Participant 1),* reflecting the frustration of navigating credentialing systems that often undervalue international education.

Many participants expressed disappointment upon realizing that their advanced degrees did not necessarily translate into immediate professional opportunities or recognition in the Canadian healthcare system. The mismatch between expectations and reality was a recurring theme, with several nurses noting that their credentials were not fully acknowledged and that they had to undergo additional training despite their qualifications. One participant shared that although she held a PhD and had taught at the graduate level abroad, she was told she needed to “go back to basics” and prepare for exams as if she were a new graduate (Participant 8).

Finally, these challenges fostered a spirit of advocacy and perseverance. Some nurses took to the media and professional networks to share their experiences and push for change. For example, one participant stated,

“I also want to advocate for nurses… especially internationally educated nurses, for nurses from different backgrounds, in terms of diversity, equity and inclusin (DEI)” (Participant 8).

##### Regulatory Requirements and Educational Gaps

Language proficiency requirements were a significant hurdle, with repeated testing and expiration of results adding financial and emotional strain. Despite having worked or studied in English-speaking environments, many IENs were still required to demonstrate their language skills through standardized exams such as IELTS or CELBAN. This led to frustration and a sense of being undervalued. One nurse expressed, *“The English exam expires every two years, it’s like your knowledge of English expires every two years. I’m like, come on. No” (Participant 5)*, highlighting how rigid policies can undermine experienced professionals.

Educational gaps identified by regulatory bodies often failed to reflect the actual competencies of IENs, especially those with advanced degrees. Bridging programs were frequently cited as both necessary and problematic. Some participants mentioned that the bridging programs were *“Blessings in disguise” (Participant 4)*. While some criticized their accessibility and effectiveness, others found them helpful for understanding Canadian healthcare systems.

##### Transition to Canadian Practice

The transition to Canadian nursing practice was often described as lengthy, confusing, and emotionally draining. Many participants encountered bureaucratic hurdles, including delays in credential recognition and unclear licensing processes. One participant noted, *“It took me 2 1/2 years just to transfer my license… they were saying that I was out of practice” (Participant 1),* highlighting the time-consuming nature of the process.

A recurring theme was the lack of transparency and support from official institutions. One participant expressed frustration: *“All the authorities, associations, everything like websites, they are pretty unclear about the process… we need to rely on other people” (Participant 3).* The absence of navigators or clear guidance from regulatory bodies was seen as a major barrier during this process. Cultural differences also played a significant role in the transition. Nurses also spoke about adjusting to new norms in patient care and professional relationships with physicians and other healthcare professionals, which illustrates the shift toward a more collaborative healthcare model in Canada.

##### Personal Supports and Challenges

Balancing family responsibilities with professional goals was a recurring challenge for many HE IENs. The demands of caregiving, especially for young children, often conflicted with the rigours of licensing and employment. One participant shared, *“It was so difficult for me to leave my kids to go to school in the morning… I feel guilty for doing that” (Participant 1)*, highlighting the emotional toll of pursuing a career while managing family life. Others described missing deadlines due to childbirth or choosing roles that accommodated parenting, underscoring the need for more flexible pathways.

Social support networks played a crucial role in helping IENs navigate the transition to Canadian practice. These networks ranged from WhatsApp groups and church communities to professional mentors and peer support in bridging programs. One nurse explained, *“We kind of like uplift each other… don’t give up and all that” (Participant 7)*, emphasizing the emotional and practical value of peer encouragement. Support from spouses, colleagues, and cultural communities also helped mitigate isolation and provided guidance through complex systems.

Despite these supports, many IENs still faced systemic and cultural barriers that left them feeling undervalued or excluded. Some described being misunderstood or judged for their cultural practices, while others noted a lack of institutional recognition for their qualifications.

##### Racism and Discrimination

Many HE IENs described experiencing racial bias and discrimination in their professional environments, often in subtle but persistent ways. Their competence was frequently questioned by patients and colleagues, leading to feelings of exclusion and a constant need to prove themselves. One nurse shared, *“I’ve been asked many times by patients… when did you start working here? It comes to me like you are questioning competence” (Participant 2)*, illustrating how assumptions based on race or accent can undermine professional credibility.

Discrimination also appeared in institutional and systemic forms, particularly in hiring practices and career progression. Some HE IENs were steered toward lower-status roles despite their qualifications, and others felt their education and experience were dismissed. These experiences reflect a broader issue of structural inequity, where immigrant professionals are undervalued despite their contributions.

Cultural scripts and interpretive frameworks further complicated the integration of IENs into Canadian healthcare settings. Nurses often felt pressure to suppress aspects of their identity to fit in, while also navigating unfamiliar norms around care and communication. One participant explained, *“I know I feel that sometimes I need to not use all my cultural background… the way I am at home… because I need to respect their perspective” (Participant 10)*.

#### Patterns of Response: Progress and Outcomes

HE IENs demonstrated varied patterns of response as they navigated the gap between expectations and realities surrounding their graduate degrees. Initially hopeful that their advanced education would lead to professional recognition, many faced disillusionment when their credentials were undervalued or even seen as barriers to employment. In response, some chose to conceal their qualifications, while others pursued Canadian graduate programs to gain legitimacy. Over time, progress was marked by emotional recovery, renewed professional identity, and strategic career moves.

##### Expectations Regarding a Graduate Degree

IENs often arrived in Canada with advanced degrees and high hopes for professional recognition and growth. However, many quickly discovered that their credentials do not carry the same weight in the Canadian healthcare system. Some even felt compelled to hide their qualifications to avoid being overlooked for jobs. The mismatch between their education and the roles they are offered leads to frustration and a sense of being underutilized.

Despite these challenges, graduate education continued to shape how others perceive HE IENs in the workplace. Colleagues and employers may assume that those with master’s or doctoral degrees possessed advanced clinical or leadership skills, which can create pressure to meet elevated expectations.

For many, pursuing further education in Canada became a strategic move to gain legitimacy and improve career prospects. One participant reflected, *“My plan is… to take my master’s again here, hopefully it will matter now… because it’s a Canadian master’s” (Participant 5)*. This sentiment underscored the belief that Canadian credentials are more valued and necessary for advancement, even among those who already held graduate degrees from abroad.

##### Overall Wellbeing

The journey to professional integration in Canada had a profound impact on the emotional well-being of HE IENs. Many described feelings of anxiety, frustration, and identity loss during the long and uncertain licensing process. The inability to practice their profession despite years of experience led to a sense of stagnation and diminished self-worth. One participant shared, *“Suddenly I was nothing because I couldn’t be a nurse here… but getting the license was OK. Now I can be a nurse” (Participant 12)*, capturing the emotional burden of regaining professional identity.

Job satisfaction played a significant role in restoring emotional well-being. Participants expressed joy and relief upon securing roles that aligned with their skills and passions. Celebrations with family and colleagues marked milestones, such as passing licensing exams or starting academic positions, reinforcing a sense of achievement and belonging.

#### Recommendations

Participants in the study shared thoughtful recommendations to improve the integration and retention of HE IENs in Canada’s healthcare system. Their insights reflected lived experiences navigating complex licensing processes, cultural transitions, and employment challenges. The suggestions emphasized the need for tailored and targeted support, clearer communication, and systemic recognition of international expertise (Table 2).

**Table 2.** List of Recommendations to Improve the Integration and Retention of HE IENs in Canada

Recommendation
Peer-led orientation and mentorship
Centralized and transparent information
Support networks and professional communities
Tailored bridging and training programs
Government-funded transition initiatives
Recognition of international credentials
Dedicated navigators and streamlined processes
Alternative pathways for HE IENs
Opportunities for advocacy and policy input
Employer awareness and appreciation

## Discussion

This study examined the lived experiences of HE IENs as they transitioned into nursing practice in Canada. Through a qualitative approach, it explored the complexities of their journeys, identified the key challenges and opportunities encountered during the transition, and emphasized the need for targeted strategies and programs tailored specifically to HE IENs.

Meleis’s Transition Theory provided a unifying interpretive lens that shaped data analysis and the integration of findings across themes (Meleis, 2017). Situational transition was evident in participants’ migration and relicensure journeys, while transition conditions—both personal (e.g., resilience, family support, prior graduate education) and systemic (e.g., regulatory opacity, credential devaluation, racism)—significantly influenced transition processes. The observed patterns of response and outcomes, including identity disruption, strategic concealment of credentials, advocacy, and gradual professional reintegration, align with and extend the theory’s emphasis on transitions as dynamic, non-linear processes. Notably, the findings reveal how advanced education simultaneously functions as both a facilitator and inhibitor of healthy transition, thereby refining Transition Theory’s application to internationally educated and highly educated nursing populations.

The results of this investigation indicated that graduate education played a pivotal role in triggering changes in the immigration and professional aspirations of HE IENs, often motivating their move to Canada in pursuit of research, teaching, and career advancement. In many cases, having graduate degrees awarded points in the immigration system facilitates the HE IENs pathway to immigration (Immigration, Refugees and Citizenship Canada, 2025). The participants also mentioned that personal factors, such as family needs, influenced their decisions to immigrate, as confirmed by Carling (2024).

Many participants had extensive work experience in both academia and practice areas before immigrating to Canada. However, most found their credentials undervalued by employers and regulatory bodies and had issues submitting educational transcripts and proof of experience. Stanhope-Goodman and colleagues (2014) highlighted that costs and anxiety related to competency assessment and submitting transcripts from overseas are challenges that can hinder and significantly delay the relicensing process for IENs. These findings are also corroborated by McGuire-Brown (2025), as the pathway for licensure is still unclear and bureaucratic for many applicants, and does not effectively recognize graduate degrees.

Moreover, HE IENs experienced frustration or dissatisfaction if they could not find opportunities that aligned with their advanced qualifications or encountered barriers to career advancement within the new healthcare system. This discrepancy between their educational attainment and professional opportunities can lead to feelings of underutilization or a sense of disconnection from their chosen field (Higginbottom, 2011).

A study conducted in Finland involving Filipino IENs found similar results (Cubelo et al., 2024). IENs found it confusing to understand the requirements imposed by the Finnish regulatory bodies to obtain their license and be allowed to work in the country. Nurses with prior work and academic experience abroad did not gain a significant advantage during their transition, as they continued to face integration difficulties throughout the recruitment process (Cubelo et al., 2024).

Participants shared that a complex interplay of facilitators and inhibitors shapes the transition into Canadian practice. One of the most prominent themes is the dual role of graduate education. On one hand, participants felt their credentials hindered their integration into the job market. Crea-Arsenio et al. (2023) found similar results, affirming that IENs are often underutilized and underemployed, despite their education and experience. These qualifications were often undervalued in employment contexts, with many nurses being labelled “overqualified” or finding that their degrees did not translate into job opportunities. This trend is similar to the experience of other immigrants in Canada. Cornellissen (2021) found that immigrants who completed a bachelor’s degree or higher in a professional nursing program outside of Canada were almost four times more likely to be overqualified (58%) in their employment than those who completed the same level of education in Canada (15%). On the other hand, advanced degrees often bolstered nurses’ confidence, academic preparedness, and advocacy skills, and supported their personal skills and resilience as they continued the process.

Participants reported that licensing standards were inconsistent across provinces and lacked transparency, creating confusion and emotional strain. Despite the advanced graduate degrees held by the participants, educational gaps were still identified by regulatory bodies. This assessment often failed to reflect actual competencies, and while bridging programs were recommended to address these gaps, their accessibility and effectiveness were questioned. These findings are consistent with the literature, as studies have shown that IENs spend a significant amount of time and financial resources attending to the requirements imposed by the regulatory bodies of different provinces, despite their significant credentials (Crea-Arsenio et al., 2023; Baumann et al., 2021; Covell et al., 2017).

Many IENs reported subtle and overt forms of racism, including having their competence questioned by patients and colleagues. These experiences contributed to feelings of exclusion and a need to suppress aspects of their cultural identity to fit into Canadian norms. The emotional labour of balancing authenticity with assimilation was a recurring theme, underscoring the need for culturally sensitive support structures. Cubelo et al. (2023) discussed the occurrence of linguistic racism as experienced by IENs in Finland. This specific type of racism has a significant effect on nurses’ mental health and professional identity, as evidenced by the results of this study. Additionally, a narrative systematic review of the literature combined 17 studies and reported that IENs often experienced explicit racism in the workplaces of their host countries. This included negative attitudes from colleagues and patients, which led them to feel unwelcome and isolated. Discrimination was also evident in various forms, including limited career advancement opportunities, unequal pay, and differential treatment based on nationality or ethnicity (Rajpoot et al., 2024).

Among the facilitators, the participants noted that personal and social support played a crucial role in facilitating the transition. Peer networks, family encouragement, and community connections provided emotional resilience and practical guidance. Participants highlighted the importance of shared experiences and mutual support, often through informal channels or church communities. Moreover, IENs were often asked to complete additional education to bridge knowledge gaps in the host country. In this study, participants regarded their experience in these programs as positive and supportive. The results of this study align with the current literature, which indicates that IENs agree that bridging programs support professional integration and development, although they also cause significant personal and financial difficulties (Balakumaran et al., 2023; Cruz et al., 2022).

### Recommendations and Implications for Practice

The participants shared several recommendations that emphasize a comprehensive and supportive approach to integrating HE IENs into the healthcare system. Key strategies include peer-led orientation and mentorship, where IENs are guided by others with similar experiences, as well as the creation of centralized platforms that offer clear information on licensing and employment. Formal support networks, particularly for individuals with graduate degrees, are encouraged to foster a sense of community and promote shared learning. Bridging programs should be tailored to address practical, cultural, and legal differences in healthcare practice. Government-funded initiatives can provide mentorship, clinical placements, and language support. Recognition of international credentials is vital, as is assigning dedicated navigators to streamline licensing and employment processes. Alternative registration pathways should be available for HE IENs, including academic-focused roles and recognition of their expertise. IENs should also be included in policy discussions to ensure their perspectives shape healthcare systems. Employers are urged to appreciate the resilience and resourcefulness that IENs bring, particularly those from low-resource settings.

### Strengths and Limitations

This paper demonstrated several notable strengths that enhanced its scholarly contribution and overall rigour. First, it addressed a clearly articulated and significant gap in the literature by focusing specifically on highly educated internationally educated nurses (HE IENs), a population often overlooked in existing research that tended to treat IENs as a homogeneous group. The study was grounded in a well-established theoretical framework—Meleis’ Transition Theory—which provided a coherent lens for examining complex, multidimensional transition experiences and strengthened both the study design and interpretation of findings. Importantly, the findings offered nuanced and balanced insights, highlighting both the barriers and facilitators encountered by HE IENs, and were supported by participant quotations that added authenticity and depth. Finally, the paper made a meaningful practical contribution by providing clear, actionable recommendations aimed at improving integration and retention, thereby increasing its relevance for policy, education, and workforce planning in the Canadian healthcare context.

Some limitations and mitigation strategies must also be acknowledged. First, the sample size of 14 participants may limit the generalizability of the findings to the broader population of HE IENs in Canada. The use of purposive and snowball sampling, although effective in reaching a specific demographic, may have introduced selection bias and excluded individuals who are less connected to professional or social networks—particularly those who are more isolated or marginalized. The reliance on virtual interviews may have further limited participation from individuals with limited access to technology or stable internet connections, potentially excluding voices from more vulnerable or hard-to-reach populations (Valerio et al., 2016).

Member checking was not undertaken in this study due to the interpretive nature of the analysis and the use of Deductive Qualitative Analysis (DQA), which emphasizes theoretical interpretation and iterative analytic refinement rather than participant validation of findings. The study prioritized methodological rigour through alternative strategies, including investigator triangulation, transparent documentation of the analytic process, and verification of transcripts to ensure data accuracy. Additionally, given the sensitive nature of participants’ experiences and the potential burden of re-engagement, the decision not to conduct member checking aligned with ethical considerations and contemporary qualitative guidance suggesting that credibility can be robustly established through multiple, complementary validation strategies.

## Conclusion

This study provided a nuanced understanding of the lived experiences of HE IENs as they transition into nursing practice in Canada. The research highlighted the complex interplay between personal aspirations, systemic barriers, and professional realities. While graduate education often served as a catalyst for immigration and a source of confidence, it was frequently undervalued within Canadian regulatory and employment systems, leading to frustration, underemployment, and emotional strain.

Nevertheless, the study also revealed the resilience and advocacy of HE IENs, who navigated these challenges with determination and support from peer networks, bridging programs, and personal communities. Their insights underscored the urgent need for more inclusive, transparent, and supportive systems that recognize international expertise and foster equitable opportunities. The implementation of targeted recommendations can support Canadian healthcare institutions in better leveraging the skills of HE IENs and promoting a more diverse and competent workforce. This research contributes to the growing discourse on global nurse migration and calls for systemic reforms that honour the contributions of internationally educated professionals.

## Supplemental Material

Supplemental Material - The Lived Experiences of Highly Educated Internationally Educated Nurses Transitioning to Practice in Canada Supplemental Material for The Lived Experiences of Highly Educated Internationally Educated Nurses Transitioning to Practice in Canada by Emilene Reisdorfer, Mona Haimour, Amina Regina Silva, Mary Asirifi, and Margaux Papadopoulos in Sage Open Nursing.
