# Supplementary material for: The Lived Experiences of Highly Educated Internationally Educated Nurses Transitioning to Practice in Canada
Source: SAGE Open Nurs. 2026 Jun 26;12:23779608261465162. doi: 10.1177/23779608261465162 (PMC13309652; doi:10.1177/23779608261465162)
Supplement: Supplemental Material - The Lived Experiences of Highly Educated Internationally Educated Nurses Transitioning to Practice in Canada [file sj-pdf-1-son-10.1177_23779608261465162.pdf]

The Lived Experiences of Highly Educated Internationally Educated Nurses (IEN) Transitioning to Practice in Canada

Interview Guide:

Part A - Research and Interview Process

☐ Start by introducing yourself and explaining the purpose of the interview.

- *My name is _______________, and I am an (Investigator/Research Assistant) with the project "The Lived Experiences of Highly Educated Internationally Educated Nurses (IEN) Transitioning to Practice in Canada."*
- *The purposes of our research project are:*
  - *To explore the lived experiences of highly educated IENs transitioning to practice in Canada.*
  - *To identify the challenges and opportunities faced by highly educated IENs during their transition to practice in Canada.*
  - *To recommend strategies and programs specific to highly educated IENs during their transition to practice in Canada.*
- *We anticipate that this interview will take approximately one hour to complete.*

☐ Assure confidentiality and anonymity if applicable.

- *All information you share during this interview will be kept confidential and anonymous as per your signed informed consent.*

☐ Request permission to record the interview and explain how the data will be used.

- *Is it okay if we start recording this interview? The interview will be transcribed by the research assistants and analyzed by the principal investigator and co-investigators.*

☐ Start recording the interview.

Part B - Background Information:

☐ Begin with some general questions to establish rapport and put the participant at ease.

- *Examples: How is the weather at your place? How is work going? What are your plans for the weekend? What have you been watching/reading lately?*

- [ ] Apply the socio-demographic questionnaire

Part C - Semi-structured Interview Guide

I will now start asking questions related to your experience in Canada. As the interview goes by, I will remind you of the focus of the questions if needed.

- [ ] **Change Triggers and Properties of Transition:**
    - [ ] Provide some background information about your initial nursing education and professional experience before you arrived in Canada;
    - [ ] Talk about your decision to move to Canada;
    - [ ] Does having a master's degree/PhD influence your decision to move to Canada?
        - [ ] What did you expect?
    - [ ] Talk about the transition to practice in Canada and any factors that influenced this experience;
    - [ ] What were some of the challenges you encountered during the transition process?
        - [ ] Are these challenges related to your master's degree/PhD?
    - [ ] What were some of the opportunities/facilitators you encountered during the transition process?
        - [ ] Are these opportunities related to your master's degree/PhD?
    - [ ] Now let me ask you about your previous education:
        - [ ] How does having a master's degree/PhD influence your transition to practice?

- [ ] **Transition Conditions: Facilitators and Inhibitors:**
    - [ ] Do you have a professional support network in Canada (for instance, other IENs, institutions, colleagues, etc)? Can you talk more about this?
        - [ ] Did you have this support before you arrived in Canada, or did you establish this network after you arrived? Please explain.

- ☐ What services or support would have been helpful during your transition to practice?
  - ☐ **Can you explore how these services or supports are similar or different from the ones needed by IENs without a master's degree/PhD?**
- ☐ Talk about any language and cultural barriers you might have faced in Canada.
  - ☐ Were they related to your master's degree/PhD?

☐ **Patterns of Response: Progress and Outcomes:**

- ☐ How did you feel when you received your RN license?
- ☐ How was the job searching process once you received your license?
  - ☐ Did having a master's degree/PhD influence your job search? Did it influence securing and being successful at a job?
  - ☐ What expectations did you feel people have regarding your educational level?
- ☐ Have you secured your target job?
- ☐ Do you have any recommendations for programs or services to help highly educated IENs successfully transition to practice?
- ☐ Talk about your plans for the future.

Part D - Closing the Interview

a. Summarize key points discussed during the interview.
    i. Previous education
    ii. Arrival in Canada
    iii. Previous education and challenges and opportunities to transition to practice
    iv. Outcomes - where are you now?
b. Allow the participant to add any final thoughts or comments.
c. Thank them for their time and participation.
d. Reiterate any next steps or follow-up actions, such as sending a summary of the interview or contacting them for further clarification.
